# A152 ASSESSING THE USE OF A VALIDATED LYNCH SCREENING QUESTIONNAIRE IN THE OUTPATIENT SETTING AND SUBSEQUENT CHANGES IN PATIENT MANAGEMENT

**DOI:** 10.1093/jcag/gwac036.152

**Published:** 2023-03-07

**Authors:** B Smith, C Macdonnell, J Yonge, C Galaport, J Telford

**Affiliations:** Gastroenterology, University of British Columbia, vancouver, Canada

## Abstract

**Background:**

Colorectal cancer (CRC) is the second most common cause of cancer related deaths in Canada. Approximately 1 in 13 males and 1 in 18 females will develop colorectal cancer in their lifetimes. As many as 10% of CRC may be associated with an inherited syndrome. Lynch Syndrome (LS) is the most common cause of inherited CRC, estimated to account for 3-4% of all CRC cases. . A study by Kastrinos et al. found that a simple, 3 item survey, identified 77% of individuals with known LS. Implementation of this questionnaire at a gastroenterology office may help identify patients at risk for LS.

**Purpose:**

To assess whether implementation of a validated questionnaire to screen for LS is feasible in an outpatient gastroenterology clinic, and if these results would change patient management by gastroenterologists.

**Method:**

Included in this study were all patients 18 years or older, who had been referred to one of the gastroenterologists at Pacific Gastroenterology Associates in Vancouver, British Columbia. Exclusion criteria included those patients less than 18 years of age, and patients previously seen by the HCP.

Each subject was asked the following three questions: (1) Do you have a first-degree relative with CRC or LS-related cancer diagnosed before age 50? (2) Have you had CRC or polyps diagnosed before age 50? (3) Do you have ≥3 relatives with CRC?. Answering yes to any question was considered a positive screen.

Gastroenterologists were initially blinded to the content of the questionnaire and results. After completion of the questionnaire and formal gastroenterology consultation, the patients who screened positive for an increased risk of LS had their results unblinded to their primary gastroenterologist. Gastroenterologists were then asked if the survey results changed their management.

**Result(s):**

A total of 655 patients were screened, with 33 (5.0%) screening positive for question 1, 71 (10.8%) screening positive for question 2, and 17 (2.6%) screening positive for question 3. In total, 106 (15.9%) of individuals surveyed screened positive indicating higher risk for hereditary colorectal cancer.

Subsequent reassessment by gastroenterologists of patients screening positive with the LS questionnaire (n=51) yielded no change to 47 patients (92%), screening with colonoscopy not originally planned for 1 patient (2%), re-consultation for further risk assessment for 2 patients (4%) and 1 referral to the Hereditary Cancer Program (2%).

**Conclusion(s):**

Utilization of a simple 3-question survey as part of regular patient intake in a gastroenterology office resulted in an increased number of individuals being identified as being high risk for Lynch syndrome, with a subsequent increase in endoscopic screening not otherwise planned, further assessment for colon cancer risk, and an increased referral to the Hereditary Cancer Program.

**Please acknowledge all funding agencies by checking the applicable boxes below:**

None

**Disclosure of Interest:**

None Declared

